# Evaluating the quality of radiomics-based studies for endometrial cancer using RQS and METRICS tools

**DOI:** 10.1007/s00330-024-10947-6

**Published:** 2024-07-16

**Authors:** Luca Russo, Silvia Bottazzi, Burak Kocak, Konstantinos Zormpas-Petridis, Benedetta Gui, Arnaldo Stanzione, Massimo Imbriaco, Evis Sala, Renato Cuocolo, Andrea Ponsiglione

**Affiliations:** 1 Dipartimento di Scienze Radiologiche ed Ematologiche, Università Cattolica del Sacro Cuore, Rome, Italy; 2 Department of Radiology, University of Health Sciences, Basaksehir Cam and Sakura City Hospital, Basaksehir, Istanbul, Turkey; 3 Dipartimento Diagnostica per Immagini, Radioterapia Oncologica ed Ematologia, Fondazione Policlinico Universitario A. Gemelli IRCCS, Rome, Italy; 4 Department of Advanced Biomedical Sciences, University of Naples “Federico II”, Naples, Italy; 5 Department of Medicine, Surgery and Dentistry, University of Salerno, Baronissi, Italy

**Keywords:** Endometrial neoplasms, Radiomics, Machine learning, Deep learning, Quality indicators

## Abstract

**Objective:**

To assess the methodological quality of radiomics-based models in endometrial cancer using the radiomics quality score (RQS) and METhodological radiomICs score (METRICS).

**Methods:**

We systematically reviewed studies published by October 30th, 2023. Inclusion criteria were original radiomics studies on endometrial cancer using CT, MRI, PET, or ultrasound. Articles underwent a quality assessment by novice and expert radiologists using RQS and METRICS. The inter-rater reliability for RQS and METRICS among radiologists with varying expertise was determined. Subgroup analyses were performed to assess whether scores varied according to study topic, imaging technique, publication year, and journal quartile.

**Results:**

Sixty-eight studies were analysed, with a median RQS of 11 (IQR, 9–14) and METRICS score of 67.6% (IQR, 58.8–76.0); two different articles reached maximum RQS of 19 and METRICS of 90.7%, respectively. Most studies utilised MRI (82.3%) and machine learning methods (88.2%). Characterisation and recurrence risk stratification were the most explored outcomes, featured in 35.3% and 19.1% of articles, respectively. High inter-rater reliability was observed for both RQS (ICC: 0.897; 95% CI: 0.821, 0.946) and METRICS (ICC: 0.959; 95% CI: 0.928, 0.979). Methodological limitations such as lack of external validation suggest areas for improvement. At subgroup analyses, no statistically significant difference was noted.

**Conclusions:**

Whilst using RQS, the quality of endometrial cancer radiomics research was apparently unsatisfactory, METRICS depicts a good overall quality. Our study highlights the need for strict compliance with quality metrics. Adhering to these quality measures can increase the consistency of radiomics towards clinical application in the pre-operative management of endometrial cancer.

**Clinical relevance statement:**

Both the RQS and METRICS can function as instrumental tools for identifying different methodological deficiencies in endometrial cancer radiomics research. However, METRICS also reflected a focus on the practical applicability and clarity of documentation.

**Key Points:**

- *The topic of radiomics currently lacks standardisation, limiting clinical implementation*.
- *METRICS scores were generally higher than the RQS, reflecting differences in the development process and methodological content*.
- *A positive trend in METRICS score may suggest growing attention to methodological aspects in radiomics research*.

## Introduction

Radiomics is the extraction of quantitative features—invisible to the human eye—from radiological images. These data, utilized independently or in conjunction with other clinical parameters, contribute to developing prediction models and support clinical decision-making [1, 2]. In the last decade, the number of studies investigating the role of radiomics in oncological imaging has exponentially grown, but its translation into clinical practice remains an unsolved issue [3–7]. Indeed, the lack of standardised methodology, reproducibility and interpretability represents the main limitations [8–12].

To address these concerns, many guidelines, checklists and scoring systems have been developed [13–17]. In 2017, the radiomics quality score (RQS) was proposed to improve the methodological rigour of the radiomic studies. Despite being originally conceived as a guide for designing appropriate radiomics studies it has since then become the “standard” evaluation tool of radiomics papers [18]. However, RQS suffers from some limitations regarding its interpretability and applicability in the assessment of deep learning studies [19]. Furthermore, it lacks an accurate description of its development process and the criteria used for assigning scores to individual items [20]. Following this initiative, several checklists have been successfully proposed to help authors and reviewers evaluate the quality of papers on the application of artificial intelligence in medicine, such as the checklist for artificial intelligence in medical imaging [15], the Must AI Criteria-10 [21] and the checklist for evaluation of radiomics research [14]. More recently, the European Society of Medical Imaging Informatics proposed the METhodological RadiomICs Score (METRICS) as an easy-to-use quality assessment tool to evaluate and improve the methodology and reproducibility of radiomics studies [20]. It includes 30 items (+ 5 conditional items) divided into nine categories and is available as a user-friendly online automated calculation tool.

Endometrial cancer is the most common gynaecological cancer in developed countries [22]. Lately, many advances have been made in understanding the complex biological behaviour of endometrial carcinoma, including the importance of molecular phenotypes in determining the prognosis [23, 24]. Furthermore, the recently revised International Federation of Gynaecologist and Obstetrics (FIGO) staging system of endometrial carcinoma now incorporates parameters exclusively accessible through biopsy and post-surgical pathological analysis, including histology, grading, lymphovascular space invasion, and molecular features [25]. Given the limitation of conventional imaging techniques in assessing these factors preoperatively, there is a growing emphasis on the role of radiomics for predictive purposes [26, 27].

In this context, we aim to critically assess the methodological quality of radiomics-based models for endometrial cancer, by means of RQS and METRICS in radiologists with different expertise.

## Methods

### Protocol

This research was conducted according to the preferred reporting items for systematic reviews and meta-analyses checklist [28].

### Search strategy

Two investigators (L.R. and S.B.) conducted a comprehensive search of PubMed and Scopus databases to identify papers published by 30th October 2023. The search string included the following terms and their variations: (“radiomics” OR “radiogenomics” OR “machine learning” OR “deep learning”) AND “endometrial cancer” AND (“computed tomography” OR “magnetic resonance” OR “positron emission tomography” OR “ultrasound”). Duplicates, non-original articles (e.g. reviews, commentaries, editorials), studies on different topics, and studies in languages other than English were removed.

### Data collection and analysis

The evaluation was carried out by four researchers, divided into pairs based on their expertise in radiomics and familiarity with the scores: two novices (L.R. and S.B.) and two expert readers (B.K. and A.P.). As an introduction to radiomics scoring systems, a preliminary training session was carried on, analysing and discussing the items of both scores. Inter-rater reliability (IRR) between reviewers was preliminarily assessed. For this task, 30 articles were randomly selected and independently reviewed by each reader [29]. Discrepancies were solved in consensus among each pair of readers and these results were considered for further analysis. For the remaining articles, each pair independently examined the components of both the RQS, made of 16 elements and the METRICS score, which includes 30 elements, with scores expressed as a percentage out of 100%. Additionally, a comprehensive analysis of the full manuscripts was conducted to extract specific data: the topic of the study (classification, characterisation, prediction of lymph node metastasis, prediction of prognosis, segmentation or treatment planning), the imaging technique (MRI, CT, US or PET/CT), the year of publication, and journal quartile.

### Statistical analysis

Qualitative variables were summarised with absolute values and percentage frequencies. The normality of the distribution of quantitative data was assessed using the Shapiro–Wilk test, and they were presented as the median and interquartile range (IQR) if not normally distributed. The intraclass correlation coefficient (ICC) was assessed with a two-way random effect, single rater, absolute agreement model based on the scores assigned by all four readers and reader pairs based on experience level on a random sample of 30 papers, based on commonly employed guidelines [29]. The resulting values were interpreted as follows: ICC < 0.5 indicated poor reliability, ICC between 0.5 and 0.75 indicated moderate reliability, ICC between 0.75 and 0.9 indicated good reliability and ICC greater than 0.90 indicated excellent reliability. A two one-sided *t*-tests (TOST) procedure was employed to compare equivalence and/or differences in the distribution of total RQS and METRICS scores, both overall and by experience group. TOST was performed with an alpha level of 0.05, assuming equivalence bounds to be between − 5% and + 5% of the total score. To determine if the overall RQS and METRICS scores differed based on the study’s objective, the type of imaging technique, the year of publication and journal quartile subgroup analyses were conducted employing the Kruskal–Wallis rank test. The statistical analysis was carried out with the use of “TOSTER” (version 0.8), “irr” (version 0.84.1), and “R” (version 4.3.2). A *p* value < 0.05 was considered statistically significant.

## Results

### Literature search

**Fig. 1:**
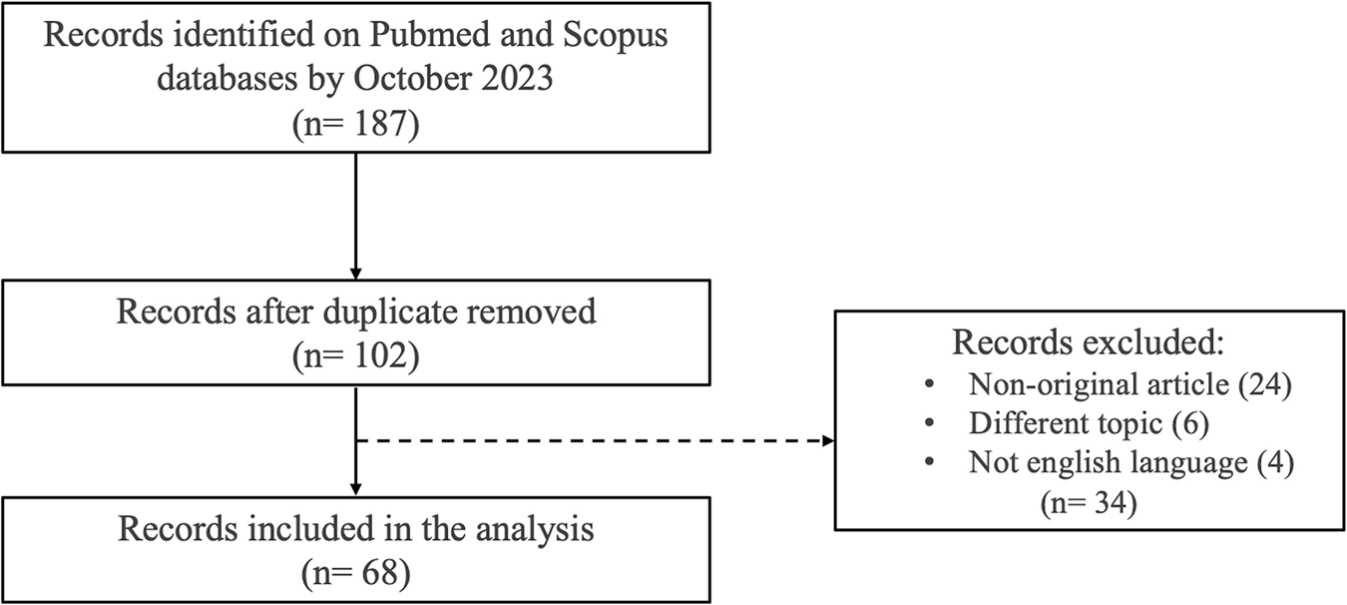
Flow diagram of literature search and selection process

The flowchart of the study selection is presented in Fig. 1. One hundred eighty-seven articles were initially identified. Of these, 85 were duplicated. Afterwards, the reviewers removed 34/102 records for the following reasons: non-original articles (24), different topics (6) and not English (4). Finally, 68 articles were included in the systematic review.

### Study characteristics

The median study population number was 171 (IQR, 138–333). Among the included papers, 24/68 (35.3%) focused on characterisation, 13/68 (19.1%) on risk stratification, 10/63 (14.7%) on classification, 9/68 (13.2%) on prediction of lymph-node metastasis, 8/68 (11.8%) on prognosis, 4/68 (5.9%) on segmentation and treatment planning (two papers each). Two out of 68 articles (2.9%) were published in 2018 and 2019 (1 each year), 8/68 (11.8%) in 2020, 19/68 (27.9%) in 2021, 18/68 (26.5%) in 2022 and 21/68 (30.9%) in 2023. MRI was the most adopted imaging modality (56/68, 82.3%), followed by CT (5/63, 7.4%), PET/CT (5/63, 7.4%) and US (2/68, 2.9%). The majority of studies used machine-learning methods (60/68, 88.2%), while deep learning was employed in 8/68 (11.8%) only.

### IRR

**Fig. 2:**
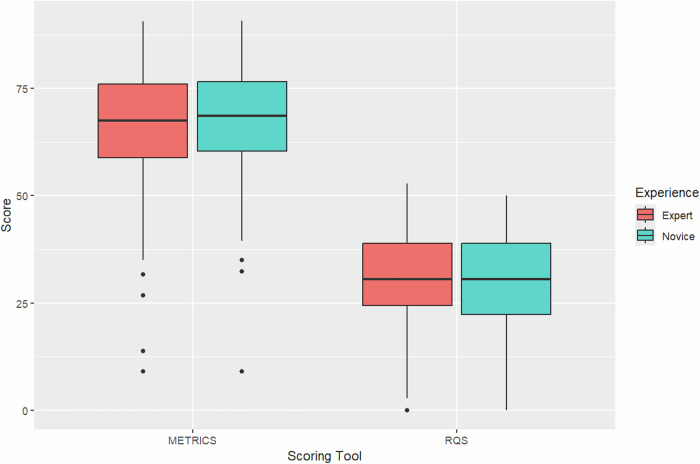
Boxplot comparison of METRICS and RQS by reviewer expertise. Scores are presented in percentage, with red boxes representing expert raters and blue boxes novice raters

ICC values indicated excellent agreement across all raters for both the RQS and METRICS scores (Fig. 2). In detail, ICC for METRICS evaluations among all raters was 0.959 (95% CI: 0.928, 0.979). For RQS evaluations, the agreement was also strong, with an ICC of 0.897 (95% CI: 0.821, 0.946), indicating a high degree of reliability among the reviewers. ICC across different pairs of readers showed excellent agreement for both METRICS (0.980, 95% CI: 0.959, 0.990) and RQS (0.940, 95% CI: 0.874, 0.971) in expert readers. For novice readers, the ICC resembled that of experts for METRICS (0.980, 95% CI: 0.958, 0.990), while it was slightly lower for RQS (0.913, 95% CI: 0.825, 0.957).

### Study evaluation—RQS

Median RQS was 11 (30.6%) for both pairs, with IQR between 9–14 (24.3–38.9%) for experts and 8–14 (22.2–38.9%) for novices. Detailed results for expert readers are reported in Table 1. The imaging protocol was well documented in 63/68 papers (92.6%), but a public protocol was used only once. Multiple segmentations were performed in 35/68 cases (51.5%). No study was conducted on phantoms or used imaging at multiple time points. In 61/68 (89.7%) studies, feature selection methods were employed. Non-radiomic variables were included in the analysis in 36/68 (52.9%), while biological correlates were detected and discussed in 12/68 (17.6%) studies. No study had a prospective design or included a cost-effectiveness analysis. A comparison with the current gold standard was performed in 40/68 cases (58.8%), whilst the potential clinical utility was analysed in 24/68 studies (35.3%). Finally, the code and/or data were available in 8/68 cases (11.8%). Code was available in 7/8 papers: for preprocessing the images and network implementation in 1 paper, for rerunning the segmentation experiments in 1 paper, for image preprocessing and data analysis in 1 paper, for data analysis in 2 papers, for radiomic data preprocessing in 1 paper, and for rerunning whole experiments in 1 paper.

**Table 1:** RQS of expert readers for all the included studies

*A*uthor (year)	Item 1	Item 2	Item 3	Item 4	Item 5	Item 6	Item 7	Item 8	Item 9	Item 10	Item 11	Item 12	Item 13	Item 14	Item 15	Item 16	RQS, (total)	RQS (%)
Bereby-Kahane M (2020)	1	0	0	0	3	0	0	1	2	0	0	− 5	0	0	0	0	2	5.6%
Bi Q (2022)	1	0	0	0	3	1	1	0	2	2	0	3	2	2	0	0	17	47.2%
Bo J (2022)	1	1	0	0	3	1	0	1	2	2	0	2	2	2	0	0	17	47.2%
Celli V (2022)	1	0	0	0	3	0	0	0	0	0	0	3	0	0	0	0	7	19.4%
Chen J (2021)	1	0	0	0	3	1	0	1	1	1	0	2	2	2	0	0	14	38.9%
Chen J (2023)	1	1	0	0	3	1	0	0	1	0	0	4	2	2	0	0	15	41.7%
Chen X (2022)	1	0	0	0	3	0	0	0	2	0	0	4	2	0	0	0	12	33.3%
K. E. Fasmer (2021)	2	1	0	0	3	0	0	0	1	1	0	2	2	0	0	0	12	33.3%
Han Y (2020)	1	1	0	0	3	0	0	0	2	0	0	− 5	2	0	0	0	4	11.1%
Hodneland E (2021)	1	1	0	0	− 3	0	0	0	1	0	0	2	2	0	0	1	5	13.9%
Hoivik EA (2021)	1	0	0	0	− 3	0	1	0	0	0	0	2	0	0	0	2	3	8.3%
Jacob H (2021)	0	0	0	0	3	0	1	0	2	0	0	2	0	0	0	0	8	22.2%
Jiang X (2023)	1	1	0	0	3	0	0	0	1	0	0	2	0	2	0	0	10	27.8%
Kurata Y (2021)	1	0	0	0	3	0	0	0	1	0	0	2	2	0	0	0	9	25.0%
Lefebvre TL (2023)	1	1	0	0	3	0	0	0	2	0	0	3	0	0	0	1	11	30.6%
Lefebvre TL (2022)	1	1	0	0	3	0	1	0	1	0	0	3	2	0	0	0	12	33.3%
Li X (2023)	1	0	0	0	3	1	0	0	2	0	0	2	0	0	0	0	9	25.0%
Li X (2023)	1	0	0	0	3	1	0	1	2	0	0	3	2	2	0	0	15	41.7%
Lin Z (2023)	1	1	0	0	3	0	0	1	1	1	0	2	0	2	0	0	12	33.3%
Lin Z (2023)	1	1	0	0	3	1	0	0	1	2	0	2	2	2	0	0	15	41.7%
Lin Z (2023)	1	1	0	0	3	1	0	1	2	1	0	5	2	2	0	0	19	52.8%
Liu D (2022)	1	1	0	0	3	1	0	0	1	2	0	2	2	2	0	0	15	41.7%
Liu J (2023)	1	1	0	0	3	1	0	1	1	1	0	3	2	2	0	0	16	44.4%
Liu XF (2022)	1	1	0	0	3	1	1	0	1	0	0	2	2	2	0	0	14	38.9%
Liu XF (2022)	1	1	0	0	3	1	1	0	1	0	0	2	2	2	0	0	14	38.9%
Liu XF (2023)	1	1	0	0	3	1	1	0	1	2	0	2	2	2	0	0	16	44.4%
Long L (2021)	1	1	0	0	3	1	0	0	1	2	0	2	0	2	0	0	13	36.1%
Luo Y (2020)	1	1	0	0	3	1	0	0	1	0	0	2	0	0	0	0	9	25.0%
Mainenti PP (2022)	1	1	0	0	3	0	0	0	1	1	0	3	0	0	0	0	10	27.8%
Miccò M (2022)	1	1	0	0	3	0	0	1	2	0	0	3	0	0	0	0	11	30.6%
Otani S (2022)	1	0	0	0	3	0	0	0	1	0	0	2	2	0	0	0	9	25.0%
Rodríguez-Ortega A (2021)	1	0	0	0	3	0	0	0	1	0	0	2	0	0	0	0	7	19.4%
Song XL (2023)	1	1	0	0	3	0	0	0	1	0	0	3	0	2	0	0	11	30.6%
Stanzione A (2021)	1	1	0	0	3	0	0	0	1	0	0	2	2	0	0	0	10	27.8%
Tan Q (2023)	1	0	0	0	3	1	0	0	1	0	0	2	0	0	0	1	9	25.0%
Wang Y (2023)	1	1	0	0	3	1	0	0	1	0	0	2	2	0	0	0	11	30.6%
Xu X (2019)	1	1	0	0	3	1	0	0	1	1	0	2	2	0	0	0	12	33.3%
Xu Y (2021)	0	0	0	0	− 3	0	0	0	1	0	0	2	0	0	0	0	0	0.0%
Yan B (2023)	1	1	0	0	3	1	0	0	1	1	0	2	0	2	0	0	12	33.3%
Yan BC (2020)	1	1	0	0	3	1	0	1	1	1	0	5	0	2	0	0	16	44.4%
Yan B (2023)	1	0	0	0	3	1	0	0	1	1	0	2	0	0	0	0	9	25.0%
Coada CA (2023)	1	1	0	0	3	0	0	0	1	0	0	− 5	0	0	0	0	1	2.8%
Crivellaro C (2020)	0	0	0	0	3	0	0	1	1	0	0	2	0	0	0	0	7	19.4%
De Bernardi E (2018)	1	0	0	0	− 3	0	0	0	1	0	0	2	2	0	0	0	3	8.0%
Huang XW (2023)	1	0	0	0	3	1	0	1	1	1	0	2	2	0	0	0	12	33.3%
Le Z (2023)	1	0	0	0	3	1	0	0	1	0	0	3	0	0	0	0	9	25.0%
Yan BC (2021)	1	1	0	0	3	0	1	0	1	0	0	4	2	0	0	0	13	36.1%
Yan BC (2021)	1	1	0	0	3	1	1	0	1	1	0	2	2	0	0	0	13	36.1%
Yang L (2023)	0	0	0	0	3	1	0	1	1	2	0	2	0	2	0	0	12	33.3%
Yang LY (2021)	1	0	0	0	3	1	0	1	1	0	0	2	2	0	0	0	11	30.6%
Yue X (2023)	1	1	0	0	3	1	0	0	1	2	0	2	2	2	0	0	15	41.7%
Zhang J (2022)	1	1	0	0	3	1	0	0	2	2	0	2	2	2	0	0	16	44.4%
Zhang K (2021)	1	1	0	0	3	1	0	0	1	1	0	2	2	2	0	0	14	38.9%
Zhang Y (2021)	1	0	0	0	3	1	0	0	0	0	0	2	0	0	0	0	7	19.4%
Zhao M (2022)	1	1	0	0	3	1	0	0	1	0	0	3	2	2	0	0	14	38.9%
Zhang K (2021)	1	1	0	0	3	1	1	1	0	0	0	2	2	0	0	0	12	33.3%
Shen L (2023)	1	0	0	0	3	0	0	0	1	0	0	2	2	0	0	0	9	25.0%
Li D (2021)	1	1	0	0	3	1	0	0	0	0	0	4	2	0	0	1	13	36.1%
Moro F (2022)	0	0	0	0	3	1	0	1	2	0	0	3	2	0	0	0	12	33.3%
Nakajo M (2021)	1	0	0	0	3	1	0	1	0	0	0	− 5	0	0	0	0	1	2.8%
Veeraraghavan H (2020)	1	0	0	0	3	1	1	0	1	0	0	2	0	0	0	2	11	30.6%
Wang X (2021)	1	0	0	0	3	0	1	1	0	1	0	3	2	2	0	1	15	41.7%
Soydal C (2022)	1	0	0	0	3	0	0	0	0	0	0	− 5	0	0	0	0	− 1	0.0%
Chen X (2020)	1	0	0	0	3	0	0	1	1	0	0	2	2	0	0	0	10	27.8%
Dong H (2020)	1	0	0	0	3	0	0	1	0	0	0	2	2	0	0	0	9	25.0%
Tao J (2022)	1	0	0	0	− 3	0	0	0	1	0	0	− 5	0	0	0	0	− 6	0.0%
Mao W (2022)	1	0	0	0	− 3	0	0	0	1	0	0	2	2	0	0	4	7	19.4%
Urushibara (2022)	1	0	0	0	− 3	0	0	0	1	0	0	2	2	0	0	0	3	8.3%

### Study evaluation—METRICS

**Fig. 3:**
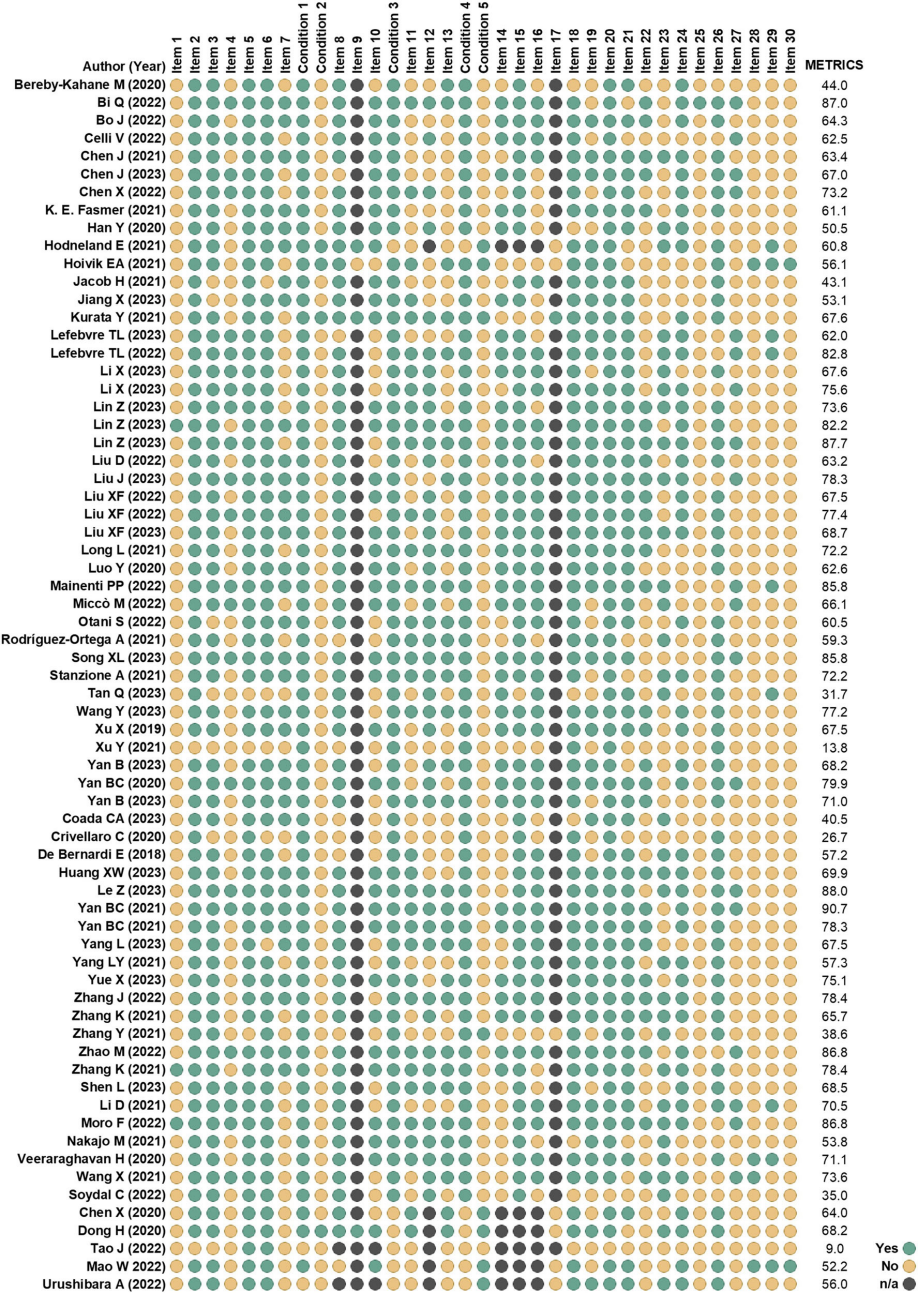
METRICS of expert readers for all the included studies

The overall median METRICS score was 67.6% (IQR, 58.8–76.0) and 68.7% (IQR, 60.4–76.5) for expert and novice readers, respectively. Figure 3 shows the results of the METRICS score for expert readers. Only 3/68 studies (4.4%) adhered to specific radiomics guidelines or checklists. The eligibility criteria of the study population and reference standard were clearly defined in 66/68 (97.1%) and 61/68 (89.7%), respectively. Clinical translatability was applicable in 65/68 studies (95.6%), and the interval between imaging used and the gold standard was clear only in 34/68 cases (50.0%). Fully automatic segmentations were performed in 4/68 studies (5.9%), with formal evaluation in 3 of them. Segmentation methods were clearly described in 57/68 (83.8%) and a single reader or automated tool produced test set segmentation in 38/68 (55.9%). Appropriate image pre-processing techniques and standardised feature extraction software were used in 36/68 (52.9%) and 43/68 (63.2%), respectively. Regarding feature selection, redundant features were removed in 57/68 (83.8%), while non-robust features were removed in 37/68 cases (54.4%). The dimensionality was appropriate to data size in 42/68 studies (61.8%). The robustness of the end-to-end pipeline was assessed in only 1/8 of studies (12.5%) employing deep learning methods. Then, metrics for evaluation of the model performance were appropriate in 66/68 studies (97.1%), including uncertainty consideration in 54/68 cases (79.4%). Uni-parametric imaging was used or proved to be inferior in 33/68 studies (48.5%). Radiomics was compared to non-radiomics approaches in 46/68 studies (67.6%), but only one compared a radiomics-based model with a simple baseline reference model. Model validation was internal in 43/68 studies (63.2%), external in 10/68 (14.7%) and both internal and external in 9/68 (13.2%), while absent in 6/68 studies (8.8%).

### Statistical analysis

**Fig. 4:**
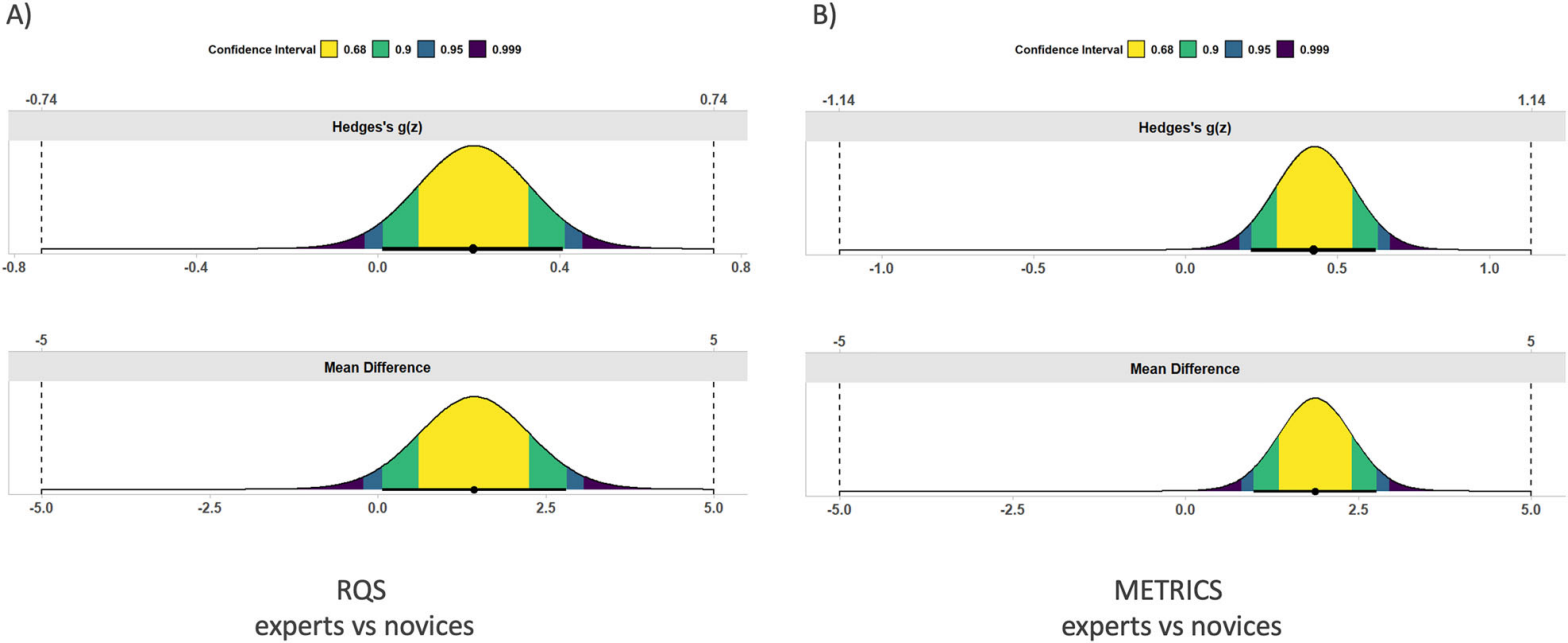
Mean difference (lower row) and effect size (upper row) plots depicting the results of the equivalence test for both RQS (**A**) and METRICS (**B**) in expert vs novice readers

**Fig. 5:**
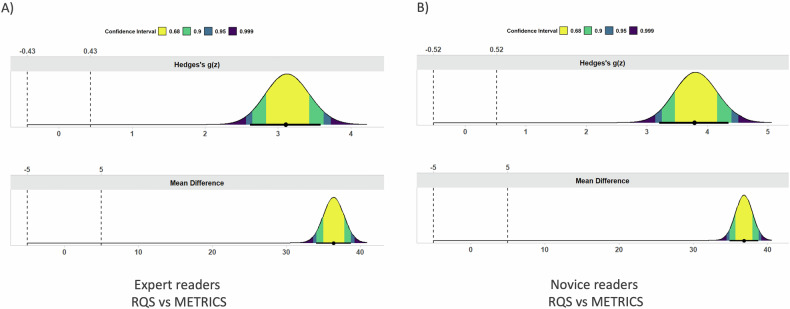
Mean difference (lower row) and effect size (upper row) plots depicting the results of the equivalence test for both expert (**A**) and novice (**B**) readers in RQS vs METRICS

The TOST procedure showed equivalence within the − 5% to 5% bounds for both RQS and METRICS in the comparison between experienced and novice readers (both *p* < 0.01). On the other hand, for both experience levels, no equivalence was found when comparing RQS, as well as METRICS total score (both *p* = 1). Figures 4 and 5 present the mean difference and effect size (i.e. Hedges’s g), as obtained by TOST. No significant differences in RQS and METRICS scores across different years of publication, imaging modalities, study topics or journal quartile. Detailed results are reported in Table 2.

**Table 2:** Subgroup analysis of endometrial cancer radiomics studies assessed by RQS and METRICS

Subgroup analysis		Number of studies	%	Median RQS, (%)	IQR, (%)	*p* value	Median METRICS, (%)	IQR, (%)	*p* value
Modality						0.13			0.13
	MRI	56	82.3	33.3	25.0–38.9		67.6	60.9–77.4	
	CT	5	7.4	25.0	13.9–33.3		70.5	36.1–79.6	
	PET/CT	5	7.4	8.0	1.4–30.6		53.8	30.9–65.4	
	US	2	2.9	33.3	33.3–33.3		78.4	69.6–NA	
Year of publication						0.14			0.17
	2023	21	30.9	33.3	25.0–41.7		69.9	67.3–77.8	
	2022	18	26.5	33.3	19.3–39.6		66.8	59.4–83.6	
	2021	19	27.9	30.6	19.4–36.1		63.4	56.1–72.2	
	2018–2020	10	14.7	25.0	10.3–31.3		63.3	48.9–68.9	
Topic						0.59			0.32
	Characterisation	24	35.3	30.6	25.0–41.0		69.9	63.0–74.7	
	Classification	10	14.7	29.2	16.3–44.4		69.5	48.8–78.3	
	Risk stratification	13	19.1	33.3	27.8–38.9		67.5	63.0–84.3	
	Prognosis and survival	8	11.8	29.2	11.8–39.6		60.8	54.4–68.2	
	Lymph-node involvement	9	13.2	31.9	10.9–38.2		60.8	40.6–67.5	
	Others	4	5.9	25.0	6.9–30.6		67.6	37.3–83.2	
Journal quartile						0.19			0.09
	First	31	45.6	28.0	25.0–36.1		71.1	60.5–82.8	
	Others	37	54.6	33.3	19.4–40.3		67.5	54.9–72.3	

The studies are divided by imaging modality, year of publication, research topic and journal quartile

## Discussion

The majority of radiomics applications were for characterisation (35.3%) [30–53], recurrence risk stratification (19.1%) [54–66] and classification [67–76]. Both RQS and METRICS scores have demonstrated excellent inter-rater reproducibility among both novice and expert readers, underscoring their reliability. This high degree of agreement can be partly ascribed to the introductory training session designed to familiarise all readers with METRICS, which is a relatively new tool compared to the more established RQS. The RQS assessment revealed an overall unsatisfactory quality, highlighting significant defects, such as the absence of phantom studies, prospective studies and cost-effectiveness analyses. Furthermore, the lack of detection and discussion of biological correlates, cut-off analysis and open-source data were noted as flaws. Interestingly, METRICS depicted an overall better quality of methodology, while still identifying weaknesses in adherence to specific radiomics and machine learning guidelines, the inclusion of fully automated segmentation processes, and limited external testing, among others.

Our findings concerning common study limitations align with previous literature, where the methodological quality of radiomics studies across various medical imaging fields has been reported as unsatisfactory by RQS application. Specifically, for endometrial cancer, Huang et al analysed MRI-based radiomics studies and reported a median total RQS percentage of 38.2%, highlighting the need for methodological improvement [77]. In other fields, the results are even less satisfactory: median total RQS percentage of 16.7%, 11.8%, 19% and 9.4% for ovarian cancer, breast cancer, meningioma and renal cell carcinoma, respectively [78–80]. To date, METRICS has just begun to be applied to radiomics studies, given its recent development.

**Fig. 6:**
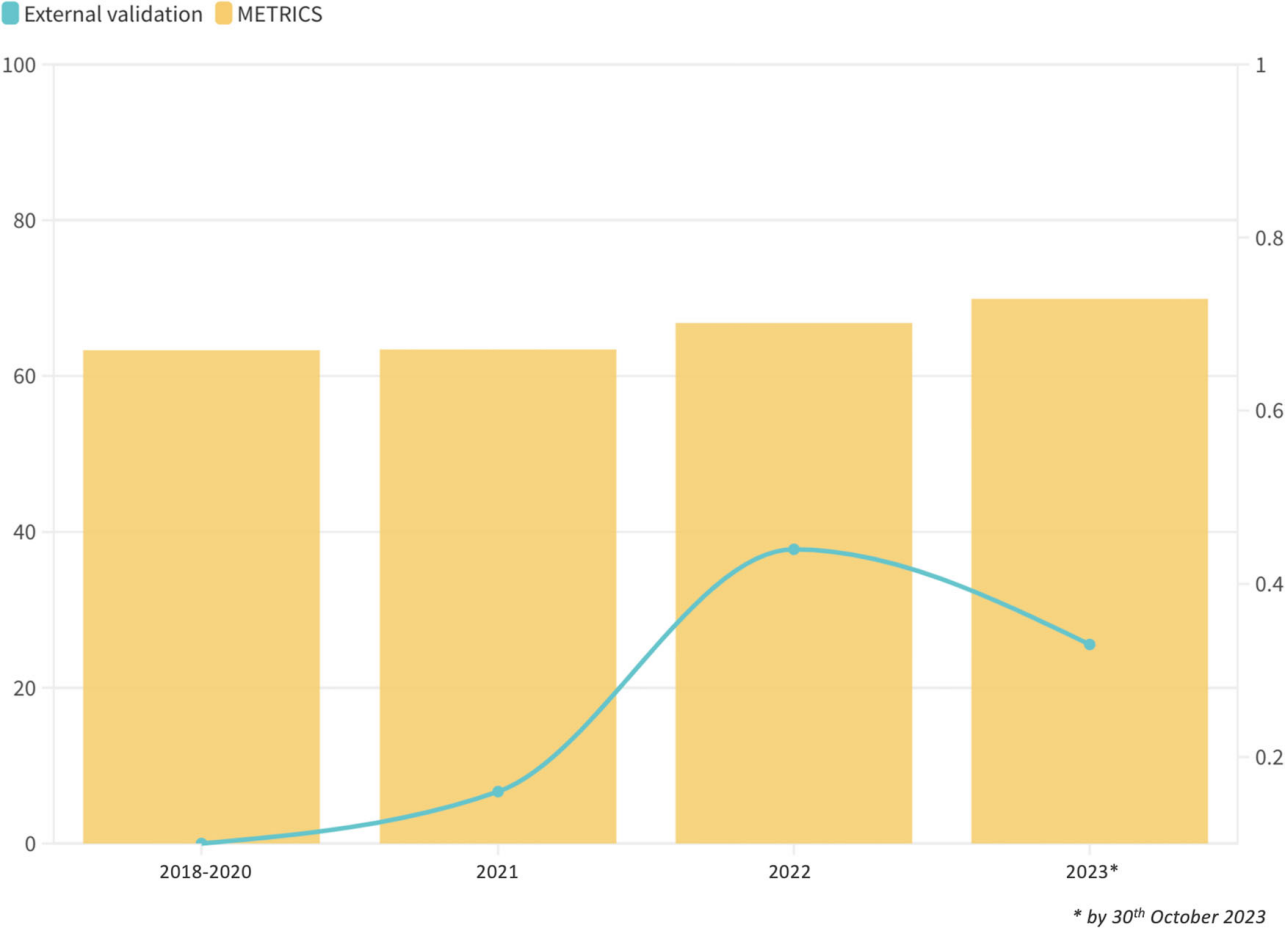
Comparative analysis of METRICS scores and external validation frequency in endometrial cancer radiomics studies from 2018 to 2023. The graph displays the annual METRICS scores (yellow bars) and the proportion of studies that included external validation (blue line)

Although the scores cannot be directly compared, when applied to deep-learning-based studies [71, 74, 76, 81–83], RQS scores these articles lower than the median compared to METRICS, due to the lack of feature selection or multiple segmentations. Furthermore, the relative weight of some items of the RQS (e.g. ± 7 points for prospective design) might be unbalanced and penalise those preliminary, exploratory studies needing retrospective design as a first ground on which stronger evidence must be built. Conversely, METRICS facilitates a step-by-step quality assessment, allowing to depict specific methodological shortcomings and, thanks to its conditional format, it permits more nuanced assessment according to methodology. One of these is the scarcity of automatic segmentation process. Despite several deep-learning-based models that have shown similar accuracy to that of experienced radiologists in segmenting endometrial cancers, automatic segmentations are far from being routinely used for radiomics studies [84]. They have shown similar accuracy to that of experienced radiologists in segmenting endometrial cancers, reducing the time needed and improving the robustness of the segmentations [82, 83]. Another lack specifically raised by METRICS is the comparison between radiomics and simple or classical statistical methods, found in one study only. This may reflect a trend towards more complex models, possibly due to the appeal of advanced machine learning for potentially superior predictive power [85]. However, comparing radiomics to simpler models is crucial [86]. It assists in establishing whether the complexity of radiomics offers significant advantages over more traditional approaches. Furthermore, cost-effectiveness and interpretability should be considered [87]. External validation has been also found to be infrequent (30.2% of studies). Although there has been a positive trend towards the years, the frequency of external validation is suboptimal, limiting the generalizability of the proposed models (Fig. 6). The discussion of biological correlates is essential for interpreting radiomics results, but infrequently depicted (17.6% of studies). In endometrial cancer, it is even more critical than in some other types of cancer because of the well-established roles of histopathological and molecular characteristics, which are now included in the FIGO staging [88, 89]. Since preoperative staging is challenging in current clinical practice, radiomics research aimed at bridging this gap must detect and discuss these biological correlates to improve its clinical translatability [90].

Overall, based on our experience, the RQS appears to skew negatively in the overall study score, reflecting an item weight distribution that is highly concentrated in a few key items. METRICS presents a generally more homogeneous item weight distribution and clearer item definitions, as well as increased flexibility in handling varying study designs. These points, partly due to the collegial, systematic development process of METRICS which may have mitigated biases by a single or a few experts, may help explain the differences in final score distribution between the two tools. In the direct comparison, as seen in Fig. 2, it appears more reasonable that the bulk of studies would hover around a moderate to good score as portrayed by METRICS rather than the low results obtained using RQS. These differences are further accentuated by the non-linear nature of the conversion from absolute to percentage score in the RQS, compared to METRICS [91].

Our review presents certain limitations. First, the rapidly evolving field of radiomics, together with variations in nomenclature, poses a risk of overlooking potential eligible studies. However, it is expected that the involvement of two reviewers has minimised this risk. Second, the chronological framework, wherein certain studies precede the adoption of the RQS and all are prior to the METRICS guidelines, could affect our evaluation [92]. Last, the necessity of conducting a training session might have affected the ICC outcomes.

To conclude, both RQS and METRICS may serve as instrumental tools in highlighting different methodological shortcomings in endometrial cancer radiomics research, that limit the interpretability and generalizability of the models. The accurate prediction of histopathological and molecular factors would preoperatively reveal the definitive FIGO stage, stratifying patients’ treatments accordingly. Notably, although being quite far from perfect, METRICS has revealed a generally satisfactory methodological quality in these studies and high reproducibility, with its scores being more accommodating, particularly for retrospective and exploratory research, reflecting its emphasis on comprehensive documentation and practical applicability. Adopting these quality scoring tools, especially METRICS, can also be used as a step-by-step guide to design radiomics studies, facilitating translatability into clinical practice.

## Supplementary information

ELECTRONIC SUPPLEMENTARY MATERIAL

## Abbreviations

FIGO: International Federation of Gynaecologist and Obstetrics
ICC: Intraclass correlation coefficient
IQR: Interquartile range
METRICS: METhodological RadiomICs Score
RQS: Radiomics quality score
TOST: Two one-sided *t*-tests
