# Supplementary material for: Evaluating the quality of radiomics-based studies for endometrial cancer using RQS and METRICS tools
Source: Eur Radiol. 2024 Jul 16;35(1):202–14. doi: 10.1007/s00330-024-10947-6 (PMC11632020; doi:10.1007/s00330-024-10947-6)
Supplement: Supplementary file 1 — ELECTRONIC SUPPLEMENTARY MATERIAL [file 330_2024_10947_MOESM1_ESM.pdf]

Evaluating the quality of radiomics based studies for endometrial cancer using RQS and METRICS tools: a systematic review
ELECTRONIC SUPPLEMENTARY MATERIAL

Supplementary Table 1. RQS of novice readers for all the included studies.

Author (Year)	Item 1	Item 2	Item 3	Item 4	Item 5	Item 6	Item 7	Item 8	Item 9	Item 10	Item 11	Item 12	Item 13	Item 14	Item 15	Item 16	RQS (Total)	RQS (%)
Bereby-Kahane M (2020)	1	0	0	0	3	0	0	0	2	0	0	-5	0	0	0	0	1	2.8
Bi Q (2022)	1	1	0	0	3	1	1	0	2	2	0	3	2	2	0	0	18	50.0
Bo J (2022)	1	1	0	0	3	1	1	1	2	1	0	2	2	2	0	0	17	47.2
Celli V (2022)	1	0	0	0	3	0	1	0	0	0	0	3	2	0	0	0	10	27.8
Chen J (2021)	1	0	0	0	3	1	0	1	1	1	0	2	2	2	0	0	14	38.9
Chen J (2023)	0	0	0	0	3	1	0	0	1	1	0	3	2	2	0	0	13	36.1
Chen X (2022)	1	0	0	0	3	0	0	0	2	0	0	3	2	0	0	0	11	30.6
K. E. Fasmer (2021)	1	0	0	0	3	0	0	1	2	0	0	2	2	0	0	0	11	30.6
Han Y (2020	1	0	0	0	3	0	0	0	0	0	0	2	2	0	0	0	8	22.2
Hodneland E (2021)	1	1	0	0	0	0	0	0	0	0	0	2	2	0	0	1	7	19.4
Hoivik EA (2021)	1	1	0	0	-3	0	1	1	0	0	0	2	0	0	0	2	5	13.9
Jacob H (2021)	0	0	0	0	3	1	0	0	2	0	0	2	0	0	0	0	8	22.2
Jiang X (2023)	0	0	0	0	3	0	0	0	1	0	0	2	2	2	0	0	10	27.8
Kurata Y (2021)	1	0	0	0	3	0	0	0	0	0	0	2	2	0	0	0	8	22.2
Lefebvre TL (2023)	1	0	0	0	3	0	0	0	2	0	0	3	0	0	0	1	10	27.8
Lefebvre TL (2022)	1	0	0	0	3	0	1	0	1	0	0	3	2	0	0	1	12	33.3
Li X (2023)	0	1	0	0	3	0	0	0	2	0	0	3	2	0	0	0	11	30.6
Li X (2023)	1	1	0	0	3	1	0	0	2	0	0	5	2	2	0	0	17	47.2
Lin Z (2023)	1	1	0	0	3	0	1	0	2	1	0	3	2	2	0	0	16	44.4
Lin Z (2023)	1	1	0	0	3	1	0	0	2	2	0	2	2	2	0	0	16	44.4
Lin Z (2023)	1	0	0	0	3	1	1	1	1	1	0	3	2	2	0	0	16	44.4
Liu D (2022)	1	0	0	0	3	1	0	0	1	2	0	2	2	2	0	0	14	38.9
Liu J (2023)	1	1	0	0	3	1	1	1	1	1	0	3	2	2	0	0	17	47.2
Liu XF (2022)	1	1	0	0	3	1	1	0	1	1	0	2	2	2	0	1	16	44.4
Liu XF (2022)	1	1	0	0	3	1	0	0	1	0	0	5	2	2	0	0	16	44.4
Liu XF (2023)	1	1	0	0	3	1	1	0	1	1	0	2	2	2	0	0	15	41.7
Long L (2021)	1	1	0	0	3	1	0	0	1	2	0	2	2	2	0	0	15	41.7
Luo Y (2020)	1	1	0	0	3	1	0	0	1	0	0	2	2	0	0	0	11	30.6
Mainenti PP (2022)	1	1	0	0	3	0	0	0	1	0	0	3	2	0	0	1	12	33.3
Miccò M (2022)	1	0	0	0	3	0	0	0	1	0	0	3	2	0	0	0	10	27.8
Otani S (2022)	1	1	0	0	3	0	0	0	2	0	0	2	2	0	0	0	11	30.6
Rodríguez-Ortega A (2021)	1	1	0	0	3	0	0	0	0	0	0	2	0	0	0	0	7	19.4
Song XL (2023)	1	1	0	0	3	0	1	0	1	0	0	3	0	2	0	0	12	33.3
Stanzione A (2021)	1	1	0	0	3	0	0	0	2	0	0	2	2	0	0	0	11	30.6
Tan Q (2023)	1	0	0	0	3	1	1	0	2	0	0	2	0	0	0	1	11	30.6
Wang Y (2023)	1	1	0	0	3	1	1	0	2	0	0	2	2	0	0	0	13	36.1
Xu X (2019)	1	1	0	0	3	1	1	0	2	1	0	2	2	0	0	0	14	38.9
Xu Y (2021)	0	0	0	0	-3	1	0	0	0	0	0	2	0	0	0	0	0	0.0
Yan B (2023)	1	1	0	0	3	1	1	0	1	1	0	2	0	2	0	0	13	36.1
Yan BC (2020)	1	1	0	0	3	1	0	0	2	1	0	5	2	2	0	0	18	50.0
Yan B (2023)	1	1	0	0	3	1	1	0	2	1	0	2	2	0	0	0	14	38.9
Coada CA (2023)	1	1	0	0	3	0	0	0	2	0	0	2	0	0	0	0	9	25.0
Crivellaro C (2020)	0	0	0	0	3	0	0	1	0	0	0	-5	2	0	0	0	1	2.8
De Bernardi E (2018)	1	0	0	0	3	0	1	1	0	0	0	2	2	0	0	0	10	27.8
Huang XW (2023)	0	0	0	0	3	1	0	0	2	0	0	2	0	0	0	0	8	22.2
Le Z (2023)	1	0	0	0	3	1	0	0	1	0	0	3	0	0	0	0	9	25.0
Yan BC (2021)	1	1	0	0	3	1	0	0	1	1	0	5	2	2	0	0	17	47.2
Yan BC (2021)	1	1	0	0	3	1	0	0	2	2	0	2	2	0	0	0	14	38.9
Yang L (2023)	0	0	0	0	3	1	0	0	1	1	0	2	2	2	0	0	12	33.3
Yang LY (2021)	1	0	0	0	-3	1	0	0	1	0	0	2	2	0	0	0	4	11.1
Yue X (2023)	1	1	0	0	3	1	0	0	1	0	0	2	2	2	0	0	13	36.1
Zhang J (2022)	1	1	0	0	3	1	0	0	1	0	0	2	2	2	0	0	13	36.1
Zhang K (2021)	1	0	0	0	3	1	0	0	1	0	0	2	2	2	0	0	12	33.3
Zhang Y (2021)	1	0	0	0	3	1	0	0	0	0	0	2	0	0	0	0	7	19.4
Zhao M (2022)	1	1	0	0	3	1	0	0	1	0	0	3	2	2	0	0	14	38.9
Zhang K (2021)	1	0	0	0	3	1	0	0	1	0	0	2	2	0	0	0	10	27.8
Shen L (2023)	1	0	0	0	3	0	0	0	1	0	0	4	2	0	0	0	11	30.6
Li D (2021)	1	1	0	0	3	1	0	0	1	0	0	3	2	0	0	1	13	36.1
Moro F (2022)	1	0	0	0	3	1	0	1	1	0	0	3	2	0	0	0	12	33.3
Nakajo M (2021)	1	0	0	0	3	1	0	0	1	0	0	-5	0	0	0	0	1	2.8
Veeraraghavan H (2020)	1	1	0	0	3	1	0	0	1	0	0	2	2	0	0	2	13	36.1
Wang X (2021)	1	0	0	0	3	0	1	1	0	0	0	3	2	2	0	1	14	38.9
Soydal C (2022)	1	0	0	0	3	0	0	0	0	0	0	-5	2	0	0	0	1	2.8
Chen X (2020)	1	1	0	0	3	0	0	1	1	0	0	2	2	0	0	0	11	30.6
Dong H (2020)	1	0	0	0	3	0	0	0	0	0	0	2	2	0	0	0	8	22.2
Tao J (2022)	1	0	0	0	-3	0	0	0	0	0	0	-5	0	0	0	0	-7	0.0
Mao W 2022)	1	0	0	0	-3	0	0	0	1	0	0	2	2	0	0	4	7	19.4
Urushibara (2022)	1	0	0	0	-3	0	0	0	1	0	0	2	2	0	0	0	3	8.3

Supplementary table 2. METRICS of novice readers for all the included studies.

Author (Year)	Item 1	Item 2	Item 3	Item 4	Item 5	Item 6	Item 7	Condition 1	Condition 2	Item 8	Item 9	Item 10	Condition 3	Item 11	Item 12	Item 13	Condition 4	Condition 5	Item 14	Item 15	Item 16	Item 17	Item 18	Item 19	Item 20	Item 21	Item 22	Item 23	Item 24	Item 25	Item 26	Item 27	Item 28	Item 29	Item 30	METRICS
Bereby-Kahane M (2020)	no	yes	yes	no	yes	yes	no	yes	no	yes	n/a	no	yes	yes	no	no	no	no	n/a	n/a	n/a	n/a	no	no	yes	yes	no	no	yes	no	no	no	no	no	no	47.6
Bi Q (2022)	no	yes	yes	yes	yes	yes	yes	yes	no	yes	n/a	yes	yes	yes	yes	yes	yes	no	yes	yes	yes	n/a	yes	no	yes	no	yes	no	yes	yes	yes	yes	no	no	no	87.0
Bo J (2022)	no	yes	yes	no	yes	yes	yes	yes	no	yes	n/a	yes	yes	yes	no	no	yes	no	yes	yes	yes	n/a	yes	yes	yes	yes	yes	no	yes	yes	yes	no	no	no	no	72.6
Celli V (2022)	no	yes	yes	yes	yes	yes	no	yes	no	yes	n/a	yes	yes	yes	yes	no	yes	no	yes	yes	yes	n/a	yes	no	yes	no	no	no	no	no	no	yes	no	no	no	69.0
Chen J (2021)	no	yes	yes	no	yes	yes	yes	yes	no	yes	n/a	yes	yes	no	no	no	yes	no	no	yes	yes	n/a	yes	yes	yes	yes	yes	yes	yes	yes	yes	no	no	no	no	65.2
Chen J (2023)	no	yes	yes	yes	yes	yes	no	yes	no	no	n/a	yes	yes	no	no	no	yes	no	yes	yes	yes	n/a	yes	yes	yes	yes	yes	no	yes	no	yes	yes	no	no	no	70.1
Chen X (2022)	no	yes	yes	yes	yes	yes	no	yes	no	yes	n/a	yes	yes	yes	yes	no	yes	no	no	yes	no	n/a	yes	no	yes	yes	no	no	yes	no	yes	yes	no	no	no	73.2
K. E. Fasmer (2021)	no	yes	yes	no	yes	yes	yes	yes	no	yes	n/a	yes	yes	no	yes	no	yes	no	yes	yes	no	n/a	yes	yes	yes	yes	no	yes	yes	no	yes	no	no	no	no	63.8
Han Y (2020	no	yes	yes	no	yes	yes	yes	yes	no	yes	n/a	yes	yes	yes	yes	no	yes	no	yes	yes	no	n/a	no	no	yes	yes	no	no	yes	no	no	no	no	no	no	55.7
Hodneland E (2021)	no	yes	yes	no	yes	yes	yes	yes	yes	yes	yes	yes	no	no	n/a	no	no	yes	n/a	n/a	n/a	no	yes	yes	yes	no	no	yes	yes	no	yes	no	no	yes	no	60.8
Hoivik EA (2021)	no	yes	yes	no	yes	yes	no	yes	yes	yes	no	yes	yes	yes	no	yes	yes	yes	no	no	no	no	yes	yes	yes	no	no	no	no	no	yes	no	yes	yes	yes	56.1
Jacob H (2021)	no	yes	no	no	yes	no	yes	yes	no	yes	n/a	yes	yes	no	no	no	yes	no	no	yes	yes	n/a	yes	yes	yes	yes	no	no	no	no	yes	no	no	no	no	43.1
Jiang X (2023)	no	yes	yes	no	yes	yes	yes	yes	no	yes	n/a	yes	yes	yes	no	no	yes	no	yes	yes	no	n/a	yes	yes	yes	yes	no	no	no	no	yes	no	no	no	no	62.7
Kurata Y (2021)	no	yes	yes	no	yes	yes	no	yes	yes	yes	yes	yes	yes	yes	yes	yes	yes	yes	yes	no	no	yes	yes	yes	yes	yes	no	no	yes	no	yes	no	no	no	no	69.6
Lefebvre TL (2023)	no	yes	yes	yes	yes	yes	no	yes	no	no	n/a	no	yes	no	yes	no	yes	no	yes	yes	no	n/a	yes	yes	yes	yes	no	yes	yes	no	no	yes	no	yes	no	65.3
Lefebvre TL (2022)	no	yes	yes	yes	yes	yes	no	yes	no	yes	n/a	no	yes	yes	yes	yes	yes	no	yes	yes	yes	n/a	yes	yes	yes	yes	no	yes	yes	no	no	yes	no	yes	no	82.8
Li X (2023)	no	yes	yes	yes	yes	yes	no	yes	no	yes	n/a	no	yes	yes	yes	no	yes	no	yes	yes	yes	n/a	yes	no	yes	yes	no	yes	no	no	yes	no	no	no	no	67.6
Li X (2023)	no	yes	yes	yes	yes	yes	no	yes	no	yes	n/a	no	yes	yes	yes	no	yes	no	yes	yes	yes	n/a	yes	yes	yes	yes	no	yes	yes	no	no	yes	no	no	no	75.6
Lin Z (2023)	no	yes	yes	yes	yes	yes	no	yes	no	yes	n/a	yes	yes	yes	yes	no	yes	no	yes	yes	no	n/a	yes	yes	yes	yes	yes	yes	yes	no	yes	no	no	no	no	73.6
Lin Z (2023)	yes	yes	yes	no	yes	yes	yes	yes	no	yes	n/a	yes	yes	yes	yes	yes	yes	no	yes	yes	yes	n/a	yes	yes	yes	yes	yes	no	yes	no	yes	no	no	no	no	82.2
Lin Z (2023)	no	yes	yes	yes	yes	yes	yes	yes	no	yes	n/a	no	yes	yes	yes	yes	yes	no	yes	yes	yes	n/a	yes	yes	yes	yes	yes	yes	yes	no	yes	yes	no	no	no	90.8
Liu D (2022)	no	yes	yes	no	yes	yes	yes	yes	no	yes	n/a	no	yes	no	yes	no	yes	no	yes	yes	no	n/a	yes	yes	yes	yes	yes	no	yes	no	yes	no	no	no	no	63.2
Liu J (2023)	no	yes	yes	yes	yes	yes	yes	yes	no	yes	n/a	yes	yes	yes	no	yes	yes	no	yes	yes	yes	n/a	yes	yes	yes	yes	yes	yes	yes	no	no	yes	no	no	no	84.8
Liu XF (2022)	no	yes	yes	no	yes	yes	yes	yes	no	yes	n/a	yes	yes	no	yes	no	yes	no	yes	yes	yes	n/a	yes	yes	yes	yes	yes	no	yes	no	yes	no	no	no	no	67.5
Liu XF (2022)	no	yes	yes	yes	yes	yes	yes	yes	no	yes	n/a	no	yes	yes	yes	no	yes	no	yes	yes	yes	n/a	yes	yes	yes	yes	yes	no	yes	no	yes	no	no	no	no	77.4
Liu XF (2023)	no	yes	yes	no	yes	yes	yes	yes	no	yes	n/a	yes	yes	no	yes	no	yes	no	yes	yes	yes	n/a	yes	yes	yes	yes	yes	yes	yes	no	yes	no	no	no	no	68.7
Long L (2021)	no	yes	yes	no	yes	yes	no	yes	no	yes	n/a	yes	yes	yes	yes	no	yes	no	yes	yes	yes	n/a	yes	yes	yes	yes	yes	no	no	no	yes	no	no	no	no	67.9
Luo Y (2020)	no	yes	yes	no	yes	yes	yes	yes	no	yes	n/a	no	yes	no	yes	no	yes	no	yes	yes	yes	n/a	yes	yes	yes	yes	no	no	no	no	yes	no	no	no	no	61.5
Mainenti PP (2022)	no	yes	yes	yes	yes	yes	yes	yes	no	yes	n/a	yes	yes	yes	yes	yes	yes	no	yes	yes	yes	n/a	yes	yes	yes	yes	yes	yes	no	no	no	yes	no	yes	no	85.8
Miccò M (2022)	no	yes	yes	yes	yes	yes	no	yes	no	yes	n/a	yes	yes	no	yes	no	yes	no	yes	yes	yes	n/a	yes	no	yes	yes	no	yes	no	no	no	yes	no	no	no	66.1
Otani S (2022)	no	yes	yes	no	yes	yes	yes	yes	no	yes	n/a	no	yes	yes	yes	yes	yes	no	no	yes	yes	n/a	yes	yes	yes	yes	no	no	yes	no	yes	no	no	no	no	73.3
Rodríguez-Ortega A (2021)	no	yes	yes	no	yes	yes	no	yes	no	no	n/a	no	yes	yes	yes	yes	yes	no	no	yes	no	n/a	yes	yes	yes	no	no	yes	no	no	yes	no	no	no	no	59.3
Song XL (2023)	no	yes	yes	yes	yes	yes	yes	yes	no	yes	n/a	yes	yes	yes	yes	no	yes	no	yes	yes	yes	n/a	yes	yes	yes	yes	no	no	no	no	yes	yes	no	no	no	81.5
Stanzione A (2021)	no	yes	yes	no	yes	yes	yes	yes	no	yes	n/a	no	yes	yes	yes	yes	yes	no	yes	yes	yes	n/a	yes	yes	yes	no	no	yes	yes	no	yes	no	no	no	no	74.1
Tan Q (2023)	no	yes	yes	no	no	no	no	yes	no	yes	n/a	no	yes	yes	yes	no	yes	no	no	no	yes	n/a	no	no	yes	yes	no	yes	no	no	yes	no	no	yes	no	45.7
Wang Y (2023)	no	yes	yes	no	yes	yes	yes	yes	no	yes	n/a	no	yes	yes	yes	yes	yes	no	yes	yes	yes	n/a	yes	yes	yes	yes	yes	no	yes	no	yes	no	no	no	no	77.2
Xu X (2019)	no	yes	yes	no	yes	yes	yes	yes	no	yes	n/a	yes	yes	no	yes	no	yes	no	yes	yes	yes	n/a	yes	yes	yes	yes	yes	no	yes	no	yes	no	no	no	no	67.5
Xu Y (2021)	no	yes	no	no	yes	yes	no	yes	no	no	n/a	no	yes	no	no	no	yes	no	no	no	no	n/a	yes	yes	yes	no	no	no	no	no	yes	no	no	no	no	32.3
Yan B (2023)	no	yes	yes	no	yes	yes	yes	yes	no	yes	n/a	yes	yes	no	no	yes	yes	no	yes	yes	yes	n/a	yes	yes	yes	yes	yes	no	yes	no	yes	no	no	no	no	68.6
Yan BC (2020)	no	yes	yes	yes	yes	yes	yes	yes	no	yes	n/a	yes	yes	yes	yes	yes	yes	no	yes	yes	yes	n/a	yes	yes	yes	yes	yes	no	yes	no	yes	yes	no	no	no	90.7
Yan B (2023)	no	yes	yes	no	yes	yes	yes	yes	no	yes	n/a	yes	yes	yes	no	yes	yes	no	yes	yes	yes	n/a	yes	no	yes	yes	yes	no	yes	no	yes	no	no	no	no	72.0
Coada CA (2023)	no	yes	yes	no	yes	yes	yes	yes	no	yes	n/a	no	yes	no	yes	no	yes	no	no	yes	no	n/a	no	no	yes	yes	no	yes	no	no	no	no	no	no	no	44.1
Crivellaro C (2020)	no	yes	yes	no	yes	no	no	yes	no	no	n/a	no	yes	no	no	no	yes	no	no	yes	yes	n/a	yes	no	yes	no	no	no	no	no	yes	no	no	no	no	39.4
De Bernardi E (2018)	no	yes	yes	no	yes	yes	no	yes	no	no	n/a	no	yes	yes	no	no	yes	no	no	yes	yes	n/a	yes	no	yes	yes	no	yes	no	no	yes	no	no	no	no	54.1
Huang XW (2023)	no	yes	yes	no	no	yes	yes	yes	no	yes	n/a	yes	yes	yes	no	no	yes	no	no	yes	yes	n/a	yes	yes	yes	yes	yes	yes	yes	no	yes	no	no	no	no	66.9
Le Z (2023)	no	yes	yes	yes	yes	yes	yes	yes	no	yes	n/a	yes	yes	yes	yes	yes	yes	no	no	yes	yes	n/a	yes	yes	yes	yes	no	yes	yes	no	yes	yes	no	no	no	88.0
Yan BC (2021)	no	yes	yes	yes	yes	yes	yes	yes	no	yes	n/a	yes	yes	yes	yes	yes	yes	no	yes	yes	yes	n/a	yes	yes	yes	yes	yes	no	yes	no	no	yes	no	no	no	86.8
Yan BC (2021)	no	yes	yes	no	yes	yes	yes	yes	no	yes	n/a	no	yes	yes	yes	yes	yes	no	yes	yes	yes	n/a	yes	yes	yes	yes	yes	no	yes	no	no	yes	no	no	no	81.1
Yang L (2023)	no	yes	yes	no	yes	no	yes	yes	no	yes	n/a	no	yes	yes	yes	yes	yes	no	no	yes	yes	n/a	yes	yes	yes	yes	yes	no	yes	no	yes	no	no	no	no	70.5
Yang LY (2021)	no	yes	yes	no	yes	yes	no	yes	no	yes	n/a	no	yes	no	no	no	yes	no	no	yes	yes	n/a	yes	yes	yes	yes	no	yes	yes	no	yes	no	no	no	no	57.3
Yue X (2023)	no	yes	yes	no	yes	yes	yes	yes	no	yes	n/a	yes	yes	yes	no	yes	yes	no	yes	yes	yes	n/a	yes	yes	yes	yes	yes	no	yes	no	yes	no	no	no	no	75.1
Zhang J (2022)	no	yes	yes	no	yes	yes	yes	yes	no	yes	n/a	yes	yes	yes	yes	yes	yes	no	yes	yes	yes	n/a	yes	yes	yes	yes	yes	no	yes	no	yes	no	no	no	no	78.3
Zhang K (2021)	no	yes	yes	no	yes	yes	no	yes	no	yes	n/a	yes	yes	no	yes	no	yes	no	yes	yes	yes	n/a	yes	yes	yes	yes	yes	yes	yes	no	yes	no	no	no	no	65.7
Zhang Y (2021)	no	yes	yes	no	no	yes	no	yes	no	no	n/a	no	yes	no	no	no	yes	yes	no	no	no	no	yes	no	yes	yes	no	yes	yes	no	yes	no	no	no	no	41.6
Zhao M (2022)	no	yes	yes	yes	yes	yes	yes	yes	no	yes	n/a	yes	yes	yes	yes	yes	yes	no	yes	yes	yes	n/a	yes	yes	yes	yes	yes	no	yes	no	yes	no	no	no	no	82.9
Zhang K (2021)	yes	yes	yes	no	yes	yes	yes	yes	no	yes	n/a	yes	yes	yes	yes	yes	yes	no	yes	yes	no	n/a	yes	yes	yes	yes	no	yes	yes	no	yes	no	no	no	no	78.4
Shen L (2023)	no	yes	yes	yes	yes	yes	no	yes	no	yes	n/a	no	yes	yes	yes	yes	yes	no	no	yes	no	n/a	yes	no	yes	yes	no	no	yes	no	yes	yes	no	no	no	76.3
Li D (2021)	no	yes	yes	yes	yes	yes	no	yes	no	yes	n/a	no	yes	no	no	no	yes	no	no	yes	yes	n/a	yes	yes	yes	yes	no	yes	yes	no	yes	yes	no	yes	no	70.5
Moro F (2022)	yes	yes	yes	yes	yes	yes	no	yes	no	yes	n/a	yes	yes	yes	yes	yes	yes	no	no	yes	yes	n/a	yes	yes	yes	yes	yes	yes	yes	no	no	yes	no	no	no	86.8
Nakajo M (2021)	no	yes	yes	no	yes	yes	no	yes	no	yes	n/a	yes	yes	yes	yes	yes	yes	no	no	yes	no	n/a	no	no	yes	no	no	yes	no	no	no	no	no	no	no	50.7
Veeraraghavan H (2020)	no	yes	yes	no	yes	yes	no	yes	no	yes	n/a	no	yes	yes	yes	yes	yes	no	no	yes	yes	n/a	yes	yes	yes	yes	no	yes	yes	no	yes	no	yes	yes	no	73.0
Wang X (2021)	no	yes	yes	yes	yes	yes	no	yes	no	yes	n/a	no	yes	no	yes	yes	yes	no	no	yes	yes	n/a	yes	yes	yes	no	yes	yes	yes	no	no	yes	yes	no	no	73.6
Soydal C (2022)	no	yes	yes	no	yes	yes	no	yes	no	yes	n/a	no	yes	no	yes	no	yes	no	no	yes	no	n/a	no	no	no	no	no	yes	no	no	no	no	no	no	no	35.0
Chen X (2020)	no	yes	yes	no	yes	yes	no	yes	no	yes	n/a	yes	yes	yes	yes	yes	yes	no	no	yes	no	n/a	no	no	yes	no	no	yes	no	no	no	no	no	no	no	50.7
Dong H (2020)	no	yes	yes	no	yes	yes	no	yes	no	yes	n/a	no	yes	yes	yes	yes	yes	no	no	yes	yes	n/a	yes	yes	yes	yes	no	yes	yes	no	yes	no	yes	yes	no	73.0
Tao J (2022)	no	no	no	no	yes	yes	no	no	no	n/a	n/a	n/a	no	no	n/a	no	no	no	n/a	n/a	n/a	n/a	no	no	no	no	no	no	no	no	no	no	no	no	no	9.0
Mao W 2022)	no	yes	yes	no	yes	yes	no	yes	yes	no	yes	yes	no	no	n/a	no	no	yes	n/a	n/a	n/a	no	yes	yes	yes	yes	no	no	yes	no	yes	no	yes	yes	yes	56.7
Urushibara (2022)	no	yes	yes	no	yes	yes	no	no	no	n/a	n/a	n/a	no	no	n/a	no	no	yes	n/a	n/a	n/a	no	yes	yes	yes	yes	no	yes	yes	no	yes	no	no	no	no	56.0
